# A dynamical framework for complex fractional killing

**DOI:** 10.1038/s41598-017-07422-2

**Published:** 2017-08-14

**Authors:** Richard Ballweg, Andrew L. Paek, Tongli Zhang

**Affiliations:** 1 Department of Molecular and Cellular Physiology, College of Medicine, University of Cincinnati, Cincinnati, OH USA; 2 Department of Molecular and Cellular Biology, University of Arizona, Tucson, AZ USA

## Abstract

When chemotherapy drugs are applied to tumor cells with the same or similar genotypes, some cells are killed, while others survive. This fractional killing contributes to drug resistance in cancer. Through an incoherent feedforward loop, chemotherapy drugs not only activate p53 to induce cell death, but also promote the expression of apoptosis inhibitors which inhibit cell death. Consequently, cells in which p53 is activated early undergo apoptosis while cells in which p53 is activated late survive. The incoherent feedforward loop and the essential role of p53 activation timing makes fractional killing a complex dynamical challenge, which is hard to understand with intuition alone. To better understand this process, we have constructed a representative model by integrating the control of apoptosis with the relevant signaling pathways. After the model was trained to recapture the observed properties of fractional killing, it was analyzed with nonlinear dynamical tools. The analysis suggested a simple dynamical framework for fractional killing, which predicts that cell fate can be altered in three possible ways: alteration of bifurcation geometry, alteration of cell trajectories, or both. These predicted categories can explain existing strategies known to combat fractional killing and facilitate the design of novel strategies.

## Introduction

Chemotherapy resistance is a major obstacle for the effective treatment of cancer. Drug resistance is found in most patients whose cancer progresses to the metastatic stage and may contribute to a poor prognosis for these patients^1^. Resistance can arise from genetic mutations or epigenetic modifications in a subpopulation of tumor cells^2, 3^. Even in a population of tumor cells with the same or similar genotypes, some tumor cells undergo apoptosis while others survive in response to chemotherapy. This observation is referred to as fractional killing^4–6^. Surviving cells may then undergo additional genetic mutations and epigenetic changes, and the progenitors of these cells may further enhance the chemotherapy resistance of the overall tumor.

To rationally design effective treatment strategies to combat fractional killing, a clear understanding of the process is essential. However, it is hard to achieve such a clear understanding by intuition alone due to the complexity of fractional killing. For example, treatment with DNA damage inducing chemotherapeutic agents has been shown to activate an incoherent feedforward loop in which the drug both promotes and represses the initiation of apoptosis. The complexity is enhanced by the essential role of p53 dynamics in controlling drug induced cell fates. Fast activation of p53 results in cellular death, while slow activation of p53 results in cell survival^5^.

To better understand the mechanisms of fractional killing in response to DNA damaging agents, we have constructed a mathematical model to represent this complex biological process. After temporal simulations of the model recaptured the observed properties of fractional killing, the model was used as an “*in-silico*” representation of fractional killing and subject to nonlinear dynamical analysis.

Dynamical analysis of the model revealed that cell fate is determined by the interplay between the temporal trajectory of p53 and CIAP and the bifurcation geometry that defines how BAXM is activated by these two control parameters. This dynamical framework might explain why tumor cells either undergo apoptosis or survive during treatment. In addition, this dynamical framework predicts that cell fate can be altered in three ways: alteration of the bifurcation geometry, alteration of the cellular trajectory, or alteration of both.

This prediction was tested against known strategies used to treat fractional killing. Three strategies, early activation of p53, flattened threshold, and reduced threshold, have been shown to effectively enhance the killing efficiency of cisplatin^5^. Each of the existing strategies for enhancing cell death fell into the predicted categories of cellular fate alteration. Based on this, novel strategies that could enhance the apoptosis of tumor cells were also designed.

## Results

### A representative model for fractional killing

Since the biological process of fractional killing cannot be analyzed directly, the current model was constructed to represent this process so that its dynamics can be analyzed. To simplify the model and facilitate its analysis, we chose to include some essential steps, but left out many detailed molecular reactions. For detailed studies of apoptosis or p53 dynamics, readers are referred to other excellent models^7–13^.

**Figure 1:**
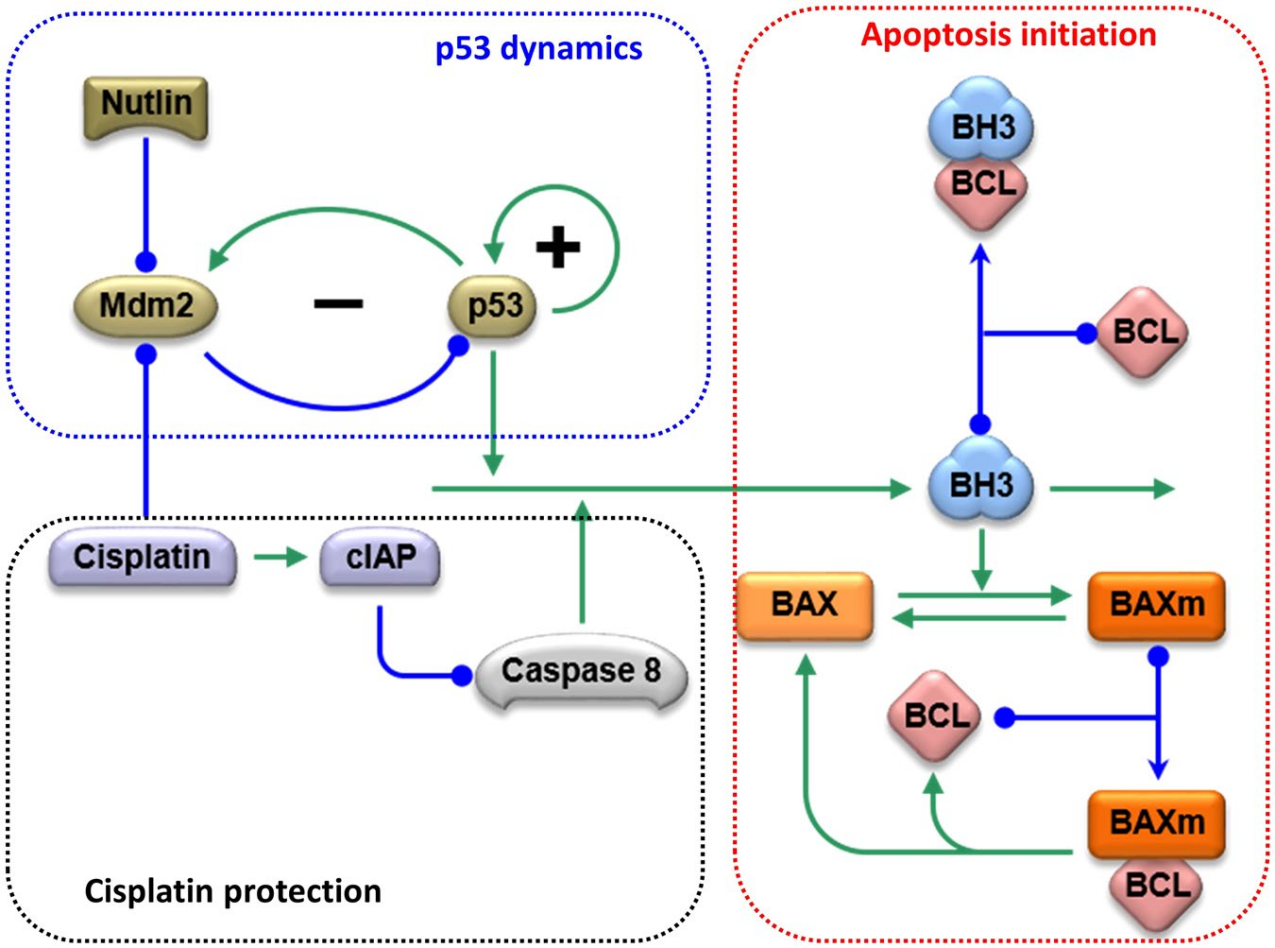
The molecular interactions in the current representative model. Nodes of different shapes represent the model components; arrows indicate activation; lines and curves with solid circle heads indicate repression. The model consists of three functional modules: p53 signaling, cisplatin signaling, and the control of BAX activation. In the p53 signaling module, p53 is controlled by one positive feedback and one Mdm2 mediated negative feedback. In the module of cisplatin signaling, cisplatin activates an incoherent feedforward loop. In one branch, cisplatin activates p53 to promote apoptosis; in the other branch, cisplatin activates CIAP to inhibit Caspase8, thus inhibiting apoptosis. In the BAX activation module, BH3 promotes the transformation of BAX from the inactive, cytoplasmic form, to the activated mitochondrial form. BAXm is inactivated from the BAXm monomer as well as the BAXm:BCL dimer.

The current model (Fig. 1) includes three modules: the initiation of apoptosis, the control of p53 dynamics, and the effect of cisplatin on these processes. Programmed cell death begins with an increase in BH3 proteins, which then bind to anti-apoptotic proteins of the BCL family. Once a sufficient amount of BH3 has accumulated, BCL proteins become saturated and free BH3 proteins are released. Free BH3 proteins induce the activation and mitochondrial translocation of BAX proteins^14–16^. The mitochondrial localized form of BAX (BAXm) can also bind to BCL, resulting in the release of additional free BH3 proteins. In this way, free BH3 and BAXm proteins activate each other forming a positive feedback. A high level of free BH3 results in additional BAXm proteins, while an increased level of BAXm proteins contributes to the release of increased free BH3 proteins.

After sufficient BAXm proteins accumulate on the mitochondrial membrane, they induce mitochondrial outer membrane permeability (MOMP)^17, 18^. MOMP results in the release of many pro-apoptotic proteins, including CTYOC, SMAC and Apoptosis Inducing Factor (AIF)^19^. SMAC binds to and inhibits the anti-apoptotic protein XIAP^20, 21^, while CytoC activates Caspase 9 to induce the execution of downstream apoptotic events. In the current model, we use BAXm activation as the indicator of apoptotic induction.

P53 is a tumor suppressor whose mutation is closely associated with tumor development. In undamaged cells, the level of p53 is kept low by Mdm2, which catalyzes the ubiquitination and degradation of p53 proteins^22^. Since Mdm2 itself is a target of the transcription factor p53, a negative feedback between p53 and Mdm2 maintains homeostasis of p53 in untreated cells^23^.

Treatment with cisplatin results in inter and intra-strand DNA crosslinks that activate the DNA damage checkpoint and disrupt the interaction between p53 and Mdm2. Interruption of the homeostasis maintaining negative feedback results in an increase in the level of p53. As p53 accumulates, it promotes apoptosis through transcriptional activation of BH3 proteins^24^. Nutlin-3, a Mdm2 inhibitor, also disrupts the negative feedback between p53 and Mdm2, inducing the accumulation of p53.

Recent work suggests that p53 is also subject to regulation by positive feedback^8, 25, 26^. Without elaborating on the detailed molecular mechanisms, the current model incorporates this positive feedback with a simple loop through which p53 activates itself.

In addition to p53, cisplatin also results in the accumulation of the anti-apoptosis protein CIAP. The increase of CIAP causes the ubiquitination and inactivation of RIP1. After the inhibition of RIP1, the formation of Ripoptosome and activation of Caspase8 are also inhibited^27, 28^. In the current representative model, the increase of CIAP is used to reduce the rate of Caspase8 activation without explicit consideration of the detailed reactions regulating the Ripoptosome.

### The model recaptures the key features of fractional killing

The model influence diagram was converted into ordinary differential equations for simulation and analysis. The equations and parameters are available in Table 1. In the Method section, we provide more elaboration on the model construction and parameter selection.

**Table 1:** Equations and parameters of the model.

Apoptosis Initiation Module			
$Bax=BaxT-BaxmT$(1)	$\frac{dBaxmT}{dt}=({k}_{f1}+{k}_{f2}\ast Bh3)\ast Bax-{k}_{b}\ast BaxmT$(2)		
$Baxm=BaxmT-Baxm:Bcl$(3)	$\frac{dBaxm:Bcl}{dt}={k}_{asXC}\ast Baxm\ast Bcl-{k}_{dsXC}\ast Baxm:bcl2-{k}_{b}\ast Baxm:Bcl$(4)		
$Bh3=Bh3T-Bh3:Bcl$(5)	$\frac{dBh3:Bcl}{dt}={k}_{asHC}\ast Bh3\ast Bcl-{k}_{dsHC}\ast Bh3:Bcl2$(6)		
**Parameters**			
${k}_{f1}=1$	${k}_{f2}=300$	${k}_{b}=2$	${k}_{asXC}=9000$
${k}_{dsXC}=0.05$	${k}_{asHC}=1000$	${k}_{dsXC}=0.01$	
**Total BH3 control**			
$$\frac{Bh3T}{dt}={k}_{sBh3}+{k}_{s2}\ast p53+{k}_{s3}\ast Caspase8-{k}_{dBH3}\ast Bh3T$$(7)			
**Parameters**			
${k}_{sBh3}=0.1$	${k}_{2}=0.2$	${k}_{3}=1$	${k}_{dBh3}=1$
**Caspase 8**			
$$\frac{dCaspase8}{dt}={k}_{aC8}-({k}_{iC8}+{k}_{i2}\ast cIAP)\ast Caspase8$$(8)			
**Parameters**			
${k}_{aC8}=0.03$	${k}_{iC8}=0.1$	${k}_{i2}=0.15$	
**CIAP**			
$$\frac{dcIAP}{dt}={k}_{sIAP}+{k}_{sIAP2}\ast \frac{Dru{g}^{n}}{Dru{g}^{n}+{J}^{n}}-{k}_{dIAP}\ast cIAP$$(9)			
**Parameters**			
$J=0.1$	${k}_{sIAP}=0.00004$	${k}_{sIAP2}=0.016$	${k}_{dIAP}=0.004$
**p53**			
$wp53={R}_{0}^{p53}+{R}_{p53}^{p53}\ast p53+{R}_{Mdm2}^{p53}\ast Mdm2$(10)		$wMdm2={R}_{0}^{wMdm2}+{R}_{p53}^{wMdm2}\ast p53$(11)	
$fp53=\frac{1}{1+{e}^{-\sigma \cdot wp53}}$(12)		$fMdm2=\frac{1}{1+{e}^{-{\sigma }_{2}\cdot wMdm2}}+\frac{1}{1+{e}^{-\sigma \ast {\rm{R}}\_drug\_effect\ast {\rm{Drug}}}}$(13)	
$\frac{dp53}{dt}=tsp53\ast (fp53-p53)$(14)		$\frac{dMdm2}{dt}=tsMdm2\ast (fMdm2-Mdm2)$(15)	
**Parameters**			
${R}_{0}^{p53}=-0.4$	${R}_{p53}^{p53}=2$	${R}_{0}^{Mdm2}=-0.55$	${R}_{p53}^{Mdm2}=1$
${R}_{Mdm2}^{p53}=-1$	$tsp53=0.6$	$tsMdm2=0.3$	$\sigma =5$
${\sigma }_{2}=10$	$n=10$		
**Initial Conditions**			
$BaxmT=0.3$	$Baxm:Bcl=0.33$	$Bh3:Bcl=0.16$	$P53=0$
$Mdm2=1$	$CIAP=0$	${R}_{\_drug\_effect}=0.33$	

**Figure 2:**
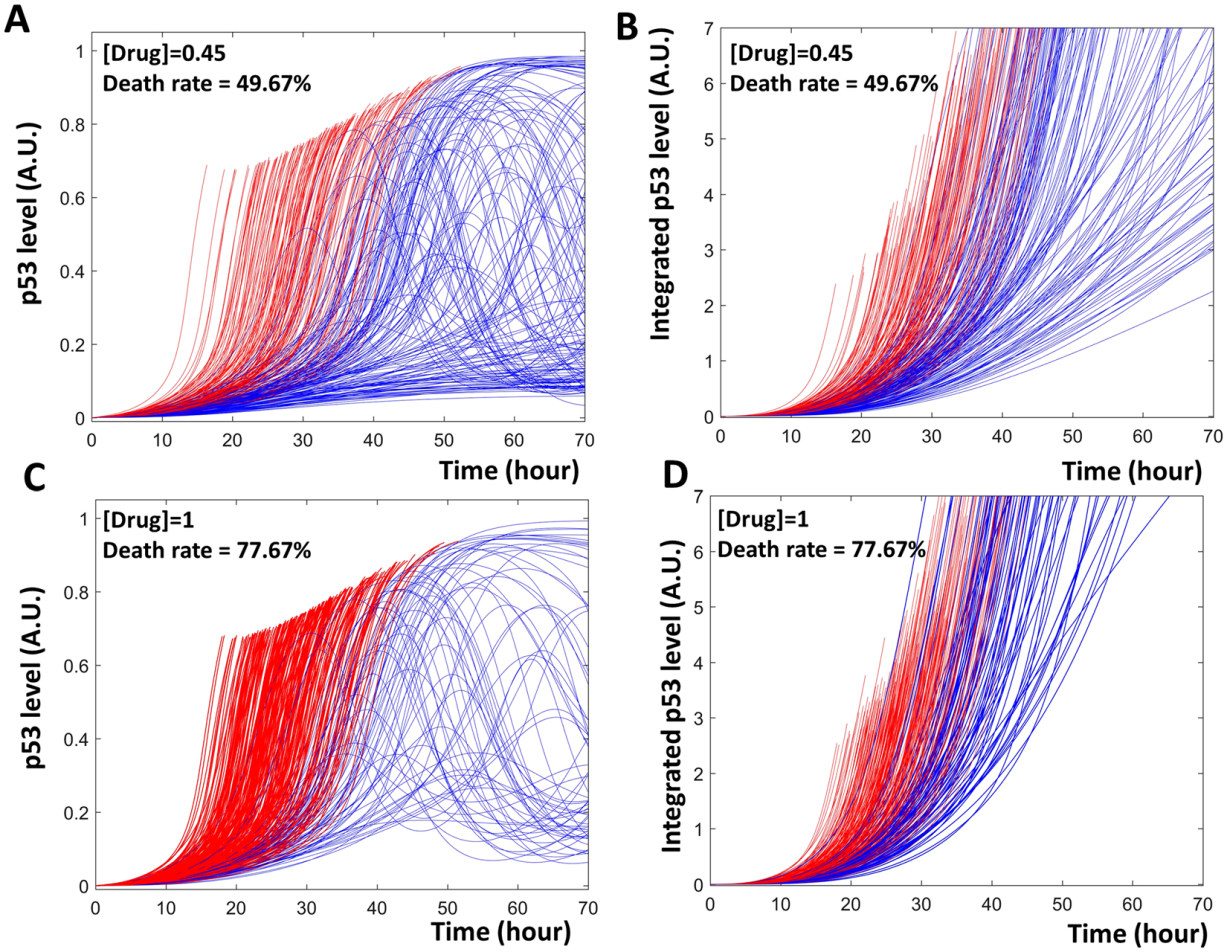
Time series simulation of fractional killing. Panels A and C show the absolute levels of p53, while panels B and D show the time integrated levels. (**A** and **B**) A total of 300 cells are subjected to a low level of drug (drug = 0.45), about half of the cells in the population quickly activate p53 and undergo apoptosis (red curves in A and B). In the remaining cells, p53 is slowly activated and these cells survive (blue curves in **A** and **B**). (**C** and **D**) When a population of 300 cells are treated with a high level of drug (drug = 1), a higher percentage of cells activate their p53 fast and undergo apoptosis (red curves in **C** and **D**).

For the model to help us understand fractional killing, it must be able to represent this complex process. We first examine whether the model recaptures experimentally observed properties of fractional killing. In response to chemotherapy drugs such as cisplatin, some cells undergo apoptosis while other cells survive and enter cell-cycle arrest until the end of the experiments. Paek *et al*. noticed that several key features characterize fractional killing of tumor cells:

1. **Similar peaks:** the maximal levels of p53 were similar in apoptotic and surviving cells (Fig. 2B of ref. 5).
2. **Different rates of p53 activation**: the activation rates of p53 show dramatic differences in the population of treated cells (Figure S1C, of ref. 5).
3. **Rate decides fate**: the rate of p53 accumulation determines whether a cell lives or dies. If p53 is activated early, the cell undergoes apoptosis; if p53 is activated late, the cell survives (Fig. 2E and F, of ref. 5).
4. **The level of integrated p53 is higher in surviving cells**: the integrated value of p53 is higher in surviving cells than in apoptotic cells (Fig. 2C, of ref. 5).
5. **Higher drug level induces more apoptosis**: if a higher level of cisplatin is used, more cells undergo apoptosis and fewer cells survive (Fig. 2G–I, of ref. 5).

To mimic the diverse dynamics of p53 in a population of cells, we chose to select random values of the parameters that control the accumulation and degradation of p53 and Mdm2 (elaborated in the Method section). Temporal simulations of the model recaptured all the key features mentioned above. In response to cisplatin, some cells activated p53 early, while others activated p53 late. Rapid activation of p53 induced apoptosis (red curves in Fig. 2A), while delayed activation of p53 was insufficient to induce apoptosis (blue curves in Fig. 2A). Peak levels of p53 were similar in all cells regardless of their fate.

When integrating the simulated p53 with respect to time, the simulation showed a higher level of integrated p53 in surviving cells (Fig. 2B). As speculated by Paek *et al*.^5^, the apoptotic cells lost their traces of p53 at cell death, while the continuous expression of p53 in surviving cells resulted in continuous accumulation of the integrated p53 value.

In response to an increase in cisplatin level, a higher percentage of cells activated p53 early and underwent apoptosis (Fig. 2C and D). As experimentally observed^5^, this higher level of cisplatin did not increase the peak values of p53.

Though simplified, the model indeed recaptured the observed features of fractional killing. Hence, it was used as an “*in silico*” representation of the process and subjected to dynamical analysis to gain additional insights into this complex process.

### Dynamical analysis revealed a simple dynamical framework for fractional killing

**Figure 3:**
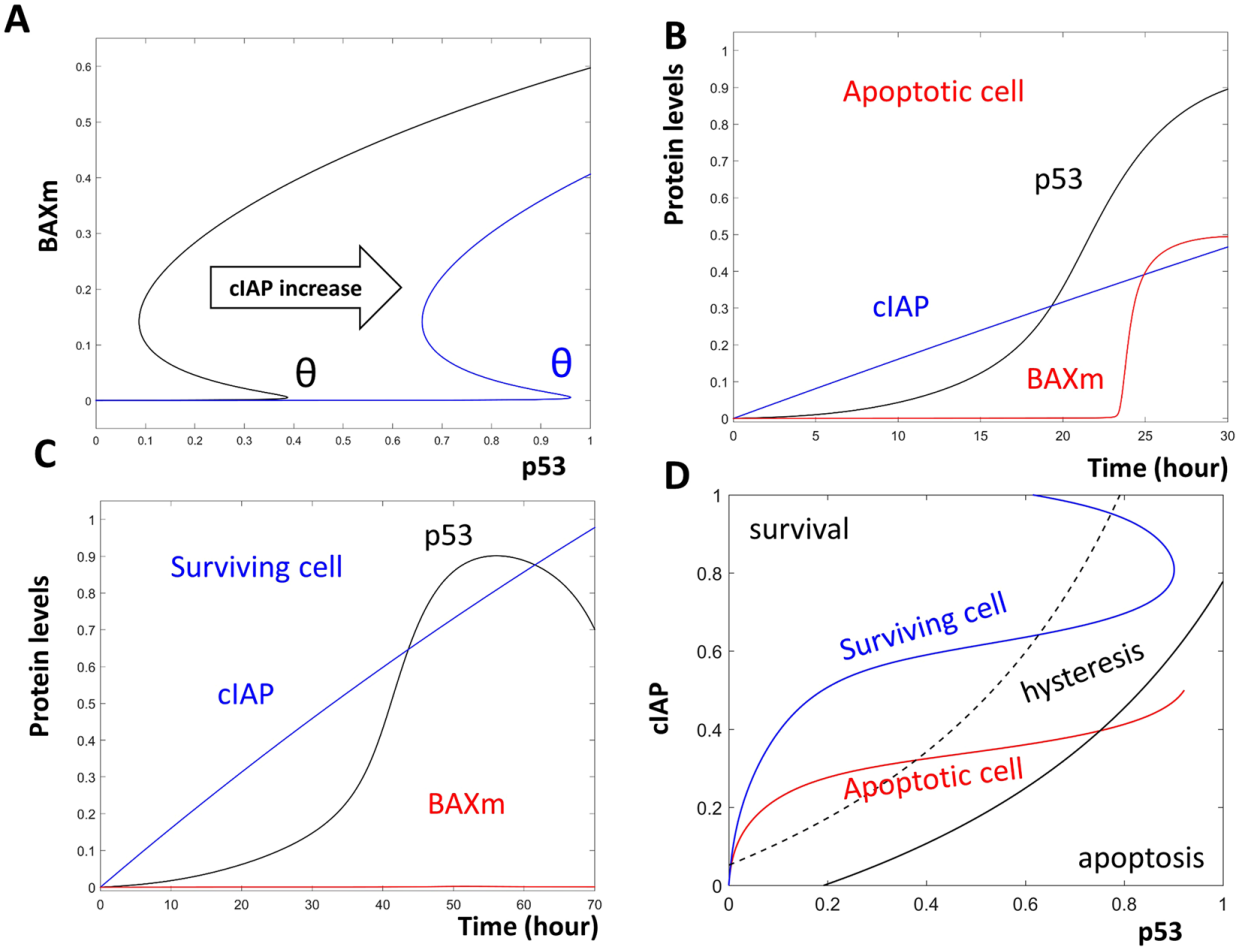
Dynamical analysis of fractional killing. (**A**) One parameter bifurcation analysis for the activation of BAXm by p53. The black curve indicates BAXm level as a function of the level of p53. At low levels of p53, BAXm is inactivated; after the level of p53 increases over the threshold (black θ), BAXm is activated. The blue curve indicates the elevated BAXm threshold (blue θ) after the level of CIAP is increased. (**B**) Time series simulation of an apoptotic cell. In this cell, p53 (black curve) is activated early, when the level of CIAP (blue curve) is low. As a result, BAXm (red curve) is activated. (**C**) Time series simulation of a surviving cell. In this cell, p53 is activated after CIAP levels have already increased. Hence, BAXm remains inactive. (**D**) Two parameter bifurcation analysis with p53 and CIAP as control parameters. The black curve indicates the threshold of BAXm activation. At the right of this black solid curve, BAXm is activated and the cell undergoes apoptosis (labelled as “apoptosis”). The black dashed curve indicates the threshold of BAXm inactivation, at the left of this dashed curve, BAXm is inactive and the cell survives (labelled as “survival”). Between the solid and the dashed curve, is the predicted hysteretic area. The red and blue curves indicate the time dependent trajectories of the apoptotic and the surviving cells, as shown in (**B** and **C**).

Using the model, we computed the level of BAXm as a function of p53. The dependence of BAXm on p53 is indicated by the black curve in Fig. 3A. At low levels of p53, the level of BAXm is low. Once the level of p53 is elevated higher than a threshold (θ in Fig. 3A), BAXm is activated and apoptosis is induced. This apoptosis initiation threshold is further regulated by the level of available CIAP in the system. If the level of CIAP is elevated, this threshold is moved rightward and a higher level of p53 is required for the system to trigger apoptosis (the blue θ in Fig. 3A). These BAXm curves are “S” shaped due to the predicted hysteresis in apoptosis initiation, which will be discussed later.

After cisplatin treatment, the level of CIAP continuously increases, as observed by Paek *et al*. (see Fig. 5A and B in ref. 5). As the available CIAP increases, there is a corresponding increase in the apoptosis initiation threshold over time. Consequently, early activation of p53 is sufficient to activate BAXm and induce apoptosis (Fig. 3B). In contrast, a similar level of p53 at a later time cannot activate BAXm due to the accumulation of CIAP (Fig. 3C).

Analysis reveals that the timing of p53 activation controls cell fate due to a time-dependent increase in the apoptosis regulator CIAP. Higher levels of CIAP result in an increase in the apoptosis initiation threshold. This control can be illustrated with a two-parameter bifurcation diagram, on which the fate of an individual cell is plotted as a function of the levels of p53 and CIAP (Fig. 3D). On this diagram, the black threshold curve traces the apoptosis initiation thresholds (the black and blue θ in Fig. 3A).

To the right of the black threshold curve, BAXm is activated and the cell undergoes apoptosis. Hence, this right area is labelled “apoptosis.” The dashed curve traces the second threshold predicted by the hysteretic switch. The region to the left of the dashed curve is labelled “survival” because cells that enter this area will not undergo apoptosis. In the area between the curves, BAXm can be either activated or inactivated depending on the previous history of the cell. Hence, this area is labelled “hysteresis” to indicate the history dependence of BAXm activity.

If p53 is activated early in a cell, the elevated p53 surpasses the apoptosis initiation threshold and pushes the cell into the “apoptosis” region, inducing cell death (the red curve in Fig. 3D corresponds to the apoptotic cell trajectory shown in Fig. 3B). However, if p53 is activated late, then increased CIAP levels raise the apoptosis threshold. Consequently, a similar level of p53 is insufficient to bring this cell through the apoptosis initiation threshold curve. Because of this, the cell survives despite having a level of active p53 similar to that in the apoptotic cell (the blue curve in Fig. 3D corresponds to the surviving cell trajectory shown in Fig. 3C).

Based on these findings, we propose a simple dynamical framework for cell fate decisions during fractional killing: cell fate is a function of the bifurcation geometry and cell trajectory. The bifurcation geometry defines distinct regions of cell survival or apoptosis. If the cell trajectory brings the cell to reside in the region corresponding to apoptosis, cell death is triggered. On the other hand, if the cell trajectory keeps the cell away from the apoptosis area, the cell survives.

### A simple dynamical framework can summarize existing strategies against fractional killing

According to this simple dynamical framework, three types of strategies can be used to enhance apoptosis and combat fractional killing: altering the bifurcation geometry, the cell trajectory, or both. We tested whether these three categories were sufficient to explain existing strategies known to enhance cell killing.

**Figure 4:**
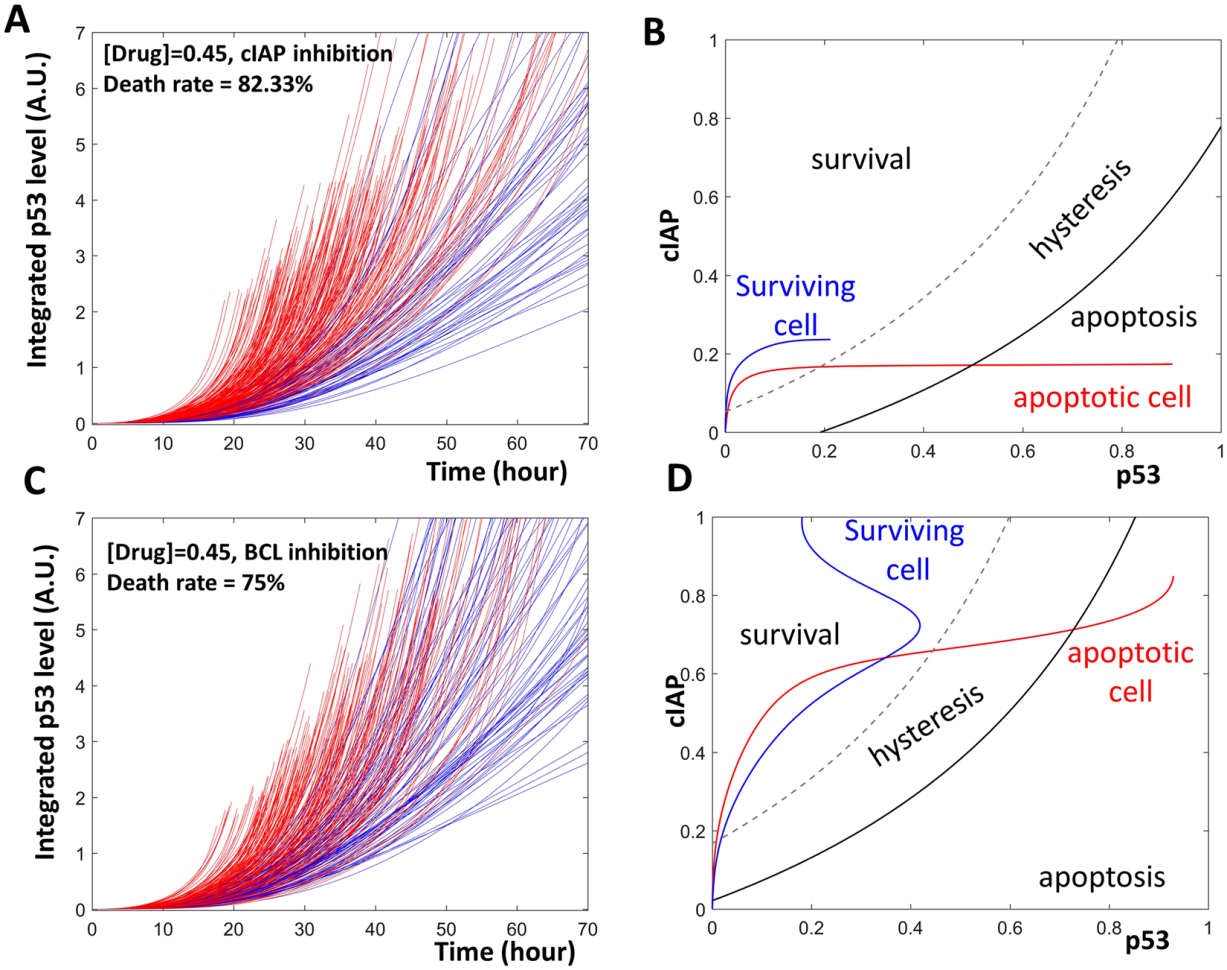
Two strategies against fractional killing. (**A**) Time series simulations of a population of cells that are treated with both cisplatin and a CIAP inhibitor. (**B**) Inhibition of CIAP changes the time dependent trajectories of an apoptotic cell (red curve) as well as a surviving cell (blue curve). (**C**) Time series simulations of a population of cells treated with cisplatin and a BCL inhibitor. (**D**) Inhibition of BCL expanded the area corresponding to apoptosis.

Treating cells with the CIAP inhibitor, LCL-161, enhances apoptosis in cells treated with cisplatin (Fig. 5F and I in ref. 5). In our model simulation, we also noticed an increased percentage of apoptotic cells when CIAP was inhibited (Figs 2A and 4A).

The time dependent trajectories of a surviving cell and an apoptotic cell were then plotted onto a two-parameter bifurcation diagram. The application of LCL-161 resulted a change in the trajectories in these cells. Since CIAP could not be activated, the cells could not move to the top region of the diagram. Rather, the cells remained in the lower portion where the apoptosis initiation threshold is low. This shows that even in a cell whose p53 activation was delayed, the activated p53 was sufficient to bring the cell over the apoptosis initiation threshold and into the region of cellular death (red curve, Fig. 4B). In the surviving cell, p53 was not sufficiently activated. The low level of p53 did not pass through the apoptosis initiation threshold, allowing the cell to avoid apoptosis and survive (blue curve, Fig. 4B).

Inhibiting BCL, another anti-apoptotic protein, resulted in a higher level of apoptosis in cisplatin treated cells (Fig. 5H and J of ref. 5). In our model, reducing the amount of available BCL also caused an increase in the percentage of apoptotic cells (Figs 2A and 4C).

**Figure 5:**
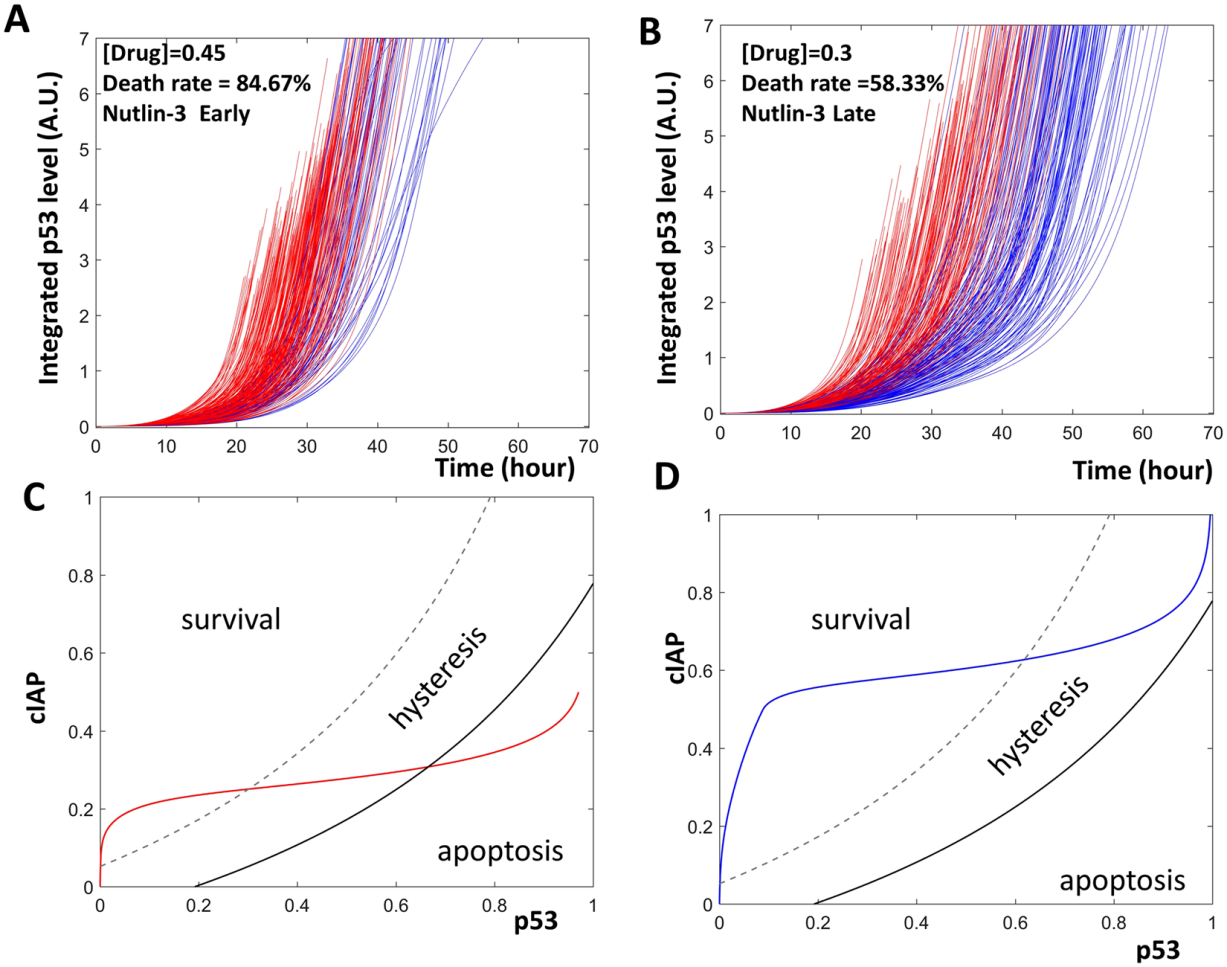
The timing of Nutlin addition controls cellular fates. (**A** and **B)** The time series simulations. The green, black, blue, and red curves indicate Nutlin, p53, CIAP and BAXm, respectively. Early Nutlin addition triggers BAXm activation (**A**). A late addition of Nutlin does not trigger the activation of BAXm (**B**). (**C** and **D**) Two parameter bifurcation diagrams together with the temporal trajectories of the apoptotic or surviving cells. Nutlin addition does not change the bifurcation geometry of the cells. Rather, an early addition of Nutlin pushes the cell trajectory into the apoptosis area (red curve, **C**), while a late Nutlin addition does not (blue curve, **D**).

Analysis of the model suggested that BCL inhibition did not alter cell trajectories, instead, the inhibition altered the bifurcation geometry (Fig. 4D). Following BCL inhibition, the apoptosis initiation threshold was shifted toward the upper-left. The shift resulted in an enlargement of the region corresponding to “apoptosis.” The enlargement results in an increase in the number of cells that were able to enter this “apoptosis” region and initiate cell death.

The p53 stabilizer Nutlin-3 has also been used to enhance apoptosis in cisplatin treated cells. Paek *et al*. noticed that cell fate after Nutlin-3 treatment was sensitive to the timing of Nutlin-3 application. If Nutlin-3 was applied early, the percentage of apoptotic cells increased; on the other hand, application of Nutlin-3 at a later time led to a minor increase in apoptosis (Fig. 3, ref. 5).

Temporal simulations of the model recaptured the apoptosis induction by early, but not late Nutlin-3 application in a population of cells (Fig. 5A and B). Furthermore, dynamical analysis suggested that the application of Nutlin-3 did not change the bifurcation geometry. Rather, the cell trajectories were altered, similar to CIAP inhibition. When Nutlin-3 was applied early, premature activation of p53 brought the cells through the low apoptosis initiation threshold, into the death region, and induced apoptosis. Alternatively, late application of Nutlin-3 activated p53 at a high apoptosis initiation threshold. Though p53 was activated and sustained at high levels, it was not sufficient to cross the already elevated threshold to trigger apoptosis (Fig. 5C and D).

Although simple, the categories predicted by the dynamical framework are sufficient to explain the existing strategies that effectively combat fractional killing. The CIAP inhibitors and Nutlin-3 work by changing cell trajectories; while BCL inhibitors work by changing the bifurcation geometries in the treated cells.

### The simple dynamical framework can facilitate the design of novel strategies to combat fractional killing

Current strategies belong to two of the three categories predicted by the simple dynamical framework: cellular trajectories are altered when CIAP is inhibited or when Nutlin-3 is added. On the other hand, the bifurcation geometry is altered when BCL is inhibited. The third potential category, that both the cellular trajectories and the bifurcation geometry can be changed simultaneously, has not been tested in previous experiments.

**Figure 6:**
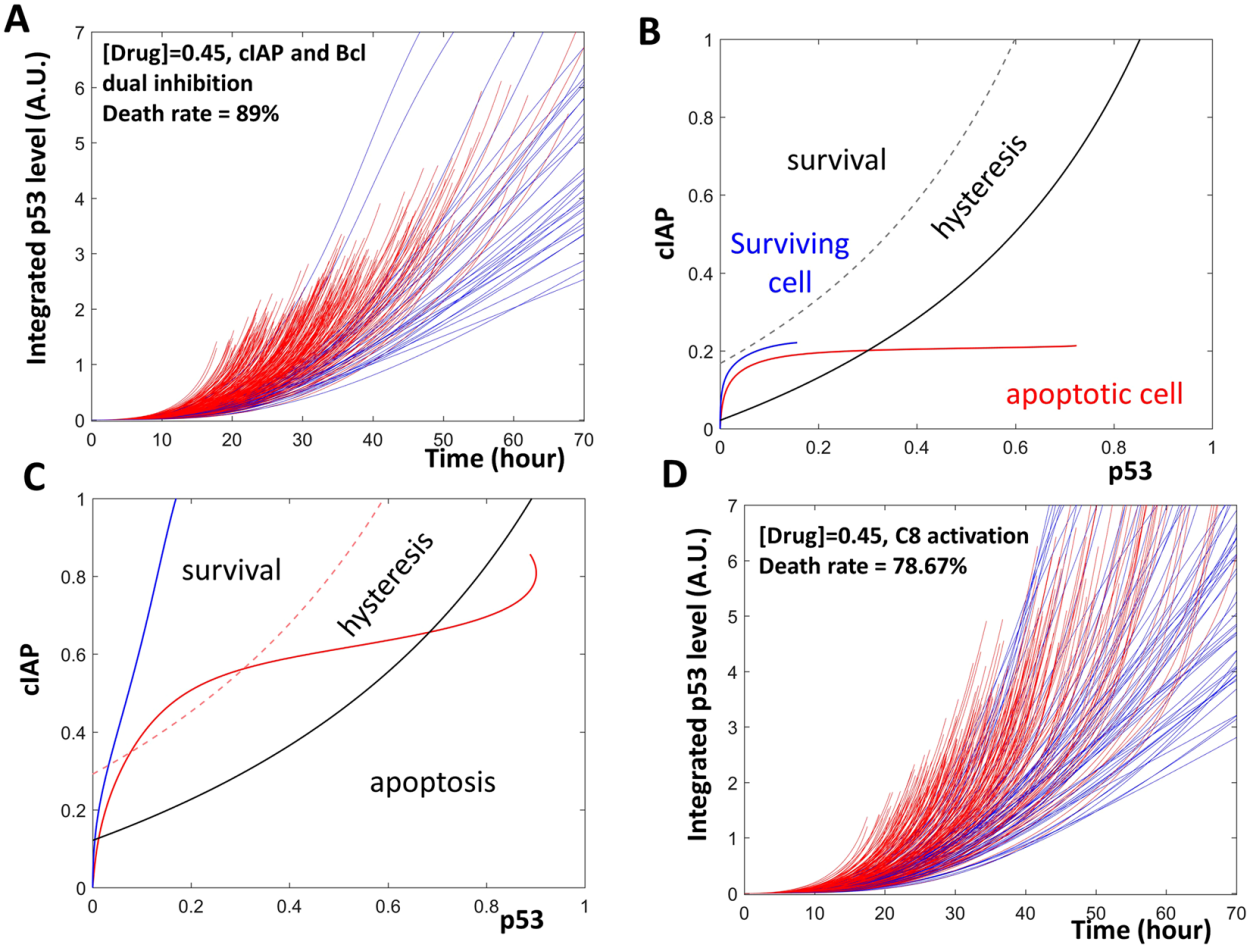
New strategies designed to combat fractional killing. (**A**) Time series simulations with a population of cells treated with cisplatin, a CIAP inhibitor, and a BCL inhibitor. (**B**) Inhibition of BCL expands the area corresponding to apoptosis; while inhibition of CIAP keeps the time dependent trajectories of the cells at the bottom of the plane. (**C**) The black solid curve and the red solid curve indicate the BAXm activation threshold before and after Caspase8 activation, respectively. The area at the right of the BAXm activation threshold curve defines the apoptosis area. Hence, the activation of Caspase8 expands the area of apoptosis. (**D**) Time series simulations with a population of cells treated with cisplatin and a Caspase8 activator.

With the model, we explored a novel approach that belongs to this third, untested category. Here, both BCL and CIAP were inhibited, resulting in alteration of both the bifurcation geometry and cellular trajectories. When compared with inhibition of either component alone, the co-inhibition resulted in a higher percentage of apoptotic cells (Fig. 6A). Nonlinear dynamical analysis of the model illustrated the synergistic effect of inhibiting these two proteins in parallel (Fig. 6B). Inhibition of BCL altered the bifurcation geometry by expanding the area of apoptosis. Meanwhile, CIAP inhibition kept the cellular trajectories at the lower part of the panel. As a result of these combined changes, more cells entered the apoptosis area and triggered cell death.

Potential novel strategies for combating fractional killing, even if they target novel proteins, can be also understood with the interplay between the bifurcation geometry and cellular trajectories. For example, if Caspase8 is activated, the curve corresponding to the apoptosis initiation threshold would move to the upper-left (black curve, Fig. 6C). This would result in the expansion of the “*apoptosis*” region. Hence, if Caspase8 activators (e.g. TRAIL ligands) are added together with cisplatin, then this strategy has the potential to enhance apoptosis, as illustrated with the simulation in a population of cells (Fig. 6D).

Given the possibility of the vast number of protein components that contribute to the decision of cell survival or cell death, the number of possible strategies is expected to be extremely large if these strategies are classified based on their target molecules. In particular, if two or more molecules are targeted, then the number of strategies will increase exponentially. In this regard, the three simple categories predicted by the dynamical framework serve as a powerful tool for us to cope with such a “combinational explosion”, to clearly classify potential strategies, and to manage these strategies effectively.

## Discussion

### Using representative models to capture the dynamical essence of complex fractional killing

**Figure 7:**
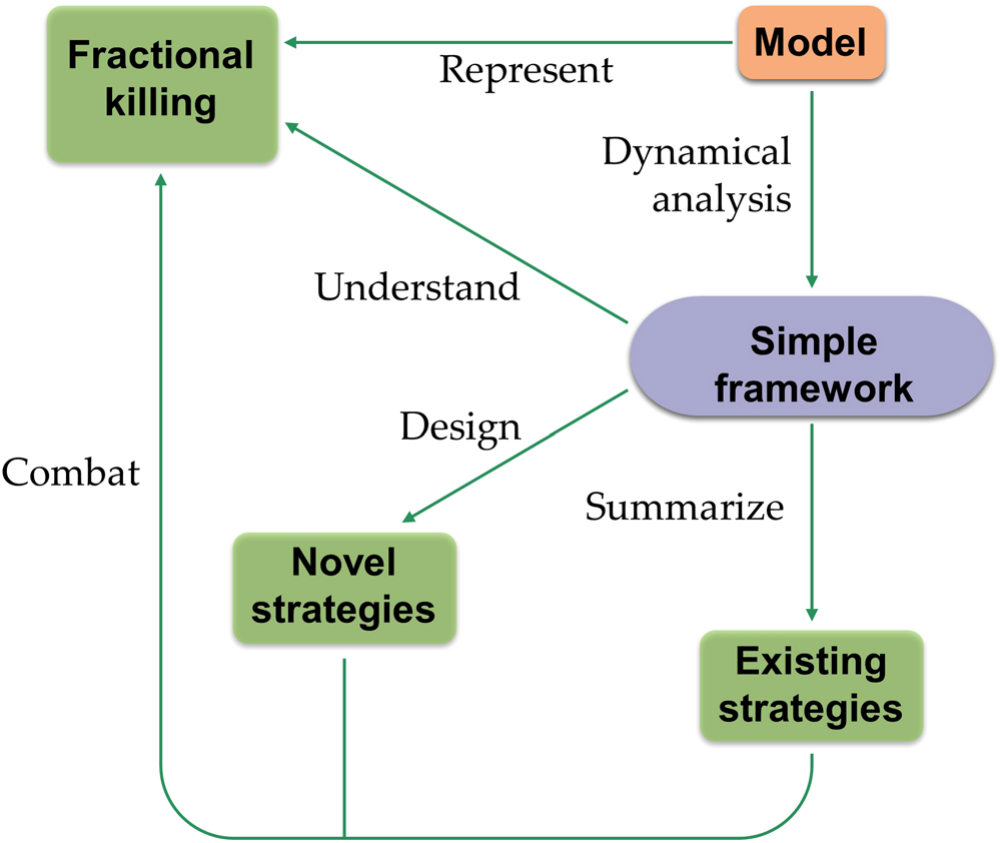
In order to understand fractional killing, a representative model is constructed and analyzed. Analysis of the model reveals a simple dynamical framework, which both explains the existing strategies and helps to design novel strategies that combat fractional killing. See discussion for elaboration.

In this study, a representative model describing the response of cancer cells to DNA damage inducing chemotherapeutic agents was used to better understand the phenomenon of fractional killing. The logical flow of the current work is summarized in Fig. 7, and we elaborate some of the key points here.

Modeling the biological system that controls drug induced fractional killing is hindered by an incomplete knowledge of the molecular mechanisms that control the phenomenon. Such limited information challenges the construction of comprehensive mechanistic models that incorporate all reaction details. In the presence of such limitations, we focus on building and analyzing representative models that recapture some of the key features of DNA damage induced fractional killing. By revealing the dynamical essence of the system, such models can give deep, meaningful insights into the design principles of fractional killing.

Temporal simulations are often sensitive to parameter values, as such it would be risky to rely on simulations alone to understand the control of fractional killing. To cope with this challenge, nonlinear dynamical tools such as phase plane analysis or bifurcation analysis have been used to explore the dynamical potential of the system for a large range of parameter values. These analyses provide us with a robust knowledge of the system’s bifurcation geometry without the need for exact parameter values.

Nonlinear analysis of the presented model revealed that the dynamical essence underpinning fractional killing is rather simple. Our model predicted that three dynamical categories should be sufficient to describe strategies that combat fractional killing. Such strategies either change the cell trajectories, bifurcation geometries, or both.

Individual cells may differ in their bifurcation geometries or their cellular trajectories. This uniqueness arises due to differences in cellular contexts, specific drugs, post-translational regulation of proteins, transcriptional regulation, stochastic gene expression, and environmental noise. However, the interplay between the bifurcation geometry and cellular trajectories would still be sufficient to understand the resulting cellular fates. Our analysis demonstrates that this simple dynamical framework is a powerful tool for understanding existing strategies and designing new ones (Table 2).

**Table 2:** List of strategies that combat fractional killing.

Strategy	Experimental result	Theoretical category*
Increasing drug concentration	Observed	CT
CIAP inhibition	Observed	CT
BCL inhibition	Observed	BG
Nutlin	Observed	CT
Inhibition of both CIAP and BCL	Prediction	AB
Caspase8 activation	Prediction	BG

*Three categories:

1. BG- alteration of bifurcation geometry;

2. CT- alteration of cellular trajectory;

3. AB- alteration of both bifurcation geometry and cellular trajectory.

### Expanding representative models to realistic ones

To construct a more comprehensive model of the molecular network that controls fractional killing, the representative model must be expanded to include additional molecular mechanisms that may control fractional killing. As an example, the pharmacokinetic and pharmacodynamic properties of cisplatin could be incorporated in to a more realistic model of the system. This expanded model will better describe how variations in cell killing result from a combination of heterogeneous drug effects and how these the differences alter the molecular network controlling p53 dynamics and BAXm activation.

The current model uses elevated BAXm level as a representative readout of cell apoptosis. In this simplification, we assume that once BAXm elevates to a specific level, the downstream caspase activation and apoptosis execution will follow^29^. Although this assumption holds for cells with functional caspases, it might not hold for cells where caspase is mutated or caspase inhibitors are overexpressed. This simplification will be addressed by incorporating the pathways downstream of MOMP in the future.

Even with an expanded model, we hypothesize that an individual cell’s fate would still be determined by the interplay between its cellular trajectory and bifurcation geometry. This may hold regardless of the drug used to treat or the control network targeted. The addition of more molecular details would expand the scope of the current model and allow it to mimic a larger, more complex molecular network. However, the essential dynamical scenarios of cell fate control would likely remain unchanged.

We plan to rigorously test this hypothesis by expanding the current model, incorporating additional molecular control details, and testing it against other known treatment strategies. If the proposal withstands this testing, then we will have an elegant but powerful tool to grasp dynamical strategies to overcome fractional killing

## Method

### A hybrid model with both generic formula and mass action kinetics

The current model uses a generic formula to describe the activation dynamics of p53 and mass activation kinetics to describe the control of apoptosis initiation. A full list of model equations and parameter values can be found in Table 1.

A generic formula is used to describe the dynamical properties of p53 without having to take into consideration of all molecular details. In this generic formula, the temporal change of each model component is modelled as a function of all other model components^30, 31^:

$$\frac{d{X}_{i}}{dt}={\tau }_{i}({F}_{i}-{X}_{i})$$

In the above formula, X_i_ represents a model component such as p53, ${{\rm{\tau }}}_{{\rm{i}}}$ is the timescale at which the steady state of X_i_ is reached and ${F}_{i}$ refers to the ratio between the steady state value of the variable ${{\rm{X}}}_{{\rm{i}}}$ and its maximum value. ${F}_{i}$ is computed as:

$${F}_{i}=\frac{1}{1+{e}^{-\sigma {W}_{i}}\,}\,$$

Here, ${F}_{i}$, has a value between zero and one and is suitable for the description of the dimensionless ratio. The parameter $\sigma$ controls the nonlinearity of the response to the net regulatory effect ${W}_{i}$, which summarizes all regulations on the variable ${{\rm{X}}}_{{\rm{i}}}$ and is computed as:

$${W}_{i}={R}_{0}^{i}+\sum _{j}{R}_{j}^{i}\cdot {X}_{j}\,$$

where ${R}_{0}^{i}$ is the background regulation and ${R}_{j}^{i}$ is the regulation exerted by model component j on model component i. The coefficient ${R}_{j}^{i}$ is negative if *j* inhibits *i*, positive if *j* activates *i*, and 0 if *j* does not regulate *i*. In this way, the values of ${R}_{j}^{i}$ are determined by the reported structure of the molecular control network.

For the model of apoptosis initiation, the details of the molecular reactions play significant roles in determining the systems dynamics. For example, the BAXm inactivation from the BAXm:BCL dimer is essential for the hysteretic activation of BAXm. In this case, mass action kinetics are suitable for describing these reaction details. The parameters of the apoptosis initiation module are modified from the previously published model^29^. Model parameters for the cellular fate control by p53 dynamics were estimated by fitting model simulations to experimental observations^5^. Robustness of parameters was examined by one parameter bifurcation analysis.

### Simulating a heterogeneous population of cells

After a population of cells were treated with cisplatin, their levels of p53 were elevated between 5 hours and 50 hours post treatment (Figure S1C, Paek *et al*.). To mimic such a heterogeneous population with dramatic differences in p53 activation times, the current model was run for multiple iterations. Because the observed p53 accumulation dynamics is different in individual cells, and many factors are known to affect these proteins^32^, the parameters controlling p53 and Mdm2 dynamics were changed within a range that covered 70–120% of the basal parameter values (Table 1) to recapture this observed heterogeneity. The 70–120% is used to represent the level of noise that causes differences between individual cells. Different noise levels were tested, and the 70–120% value yields simulation results that best recapture the experimental observations (Supplementary Figure 1).

After cisplatin treatment, most of the cells that activated p53 early underwent apoptosis; and most of the cells that activated p53 late survived. However, some exceptions were also noted. Exceptions included cells that survive despite early p53 activation and cells that undergo apoptosis even though p53 is activated late (Fig. 2G–I, ref. 5). To recapture the observed exceptions, the current model also selected the CIAP degradation randomly (70–120% of the basal value) when running for multiple iterations. It is worth noting that the stochastic nature of the molecular reactions might also contribute to cell fate decisions. Such stochastic effects have not been addressed by the variation of parameters as done in the current model, and will be addressed in future models.

All cells started with inactivated p53 and low levels of CIAP, corresponding to resting cells that experience little damage signals. The pharmacokinetic properties of cisplatin (e.g. how the drug enters the cells) as well as the pharmacodynamics parameters (e.g. how rapid DNA damage is generated and repaired in response to the drug) have also been incorporated into this model. The effects of these additional steps are lumped together in the current simplified model with the parameter, *R_drug_effect*. To pool together the heterogeneous effects of cisplatin the paramater randomly assigned (70–120% of the basal value) in individual cells.

### Plotting the trajectories of representative cells onto the bifurcation diagrams

To illustrate how the interplay between the temporal trajectory and bifurcation geometry determines the fate of an individual cell, we have plotted the trajectories of individual cells onto the bifurcation diagram. To keep the figures simple and clear, we have chosen to plot only representative cells. The fates of other cells within the simulated populations will follow patterns similar to that of the representative cells.

### Time series simulation and dynamical analysis of the model

All model equations and parameters from Table 1 were used to compute time series simulations. Settings for drug and genetic perturbations are specified in the figure legends. For one parameter bifurcation analysis, the level of p53 or the level of BH3 proteins was used as the control parameter, and the activity of BAXm was computed. For two parameter bifurcation analysis, the level of both p53 and CIAP were continuously altered, and the positions of the apoptosis initiation threshold (as well as the BAXm inactivation threshold) were computed. Computations were done using XPPAUT (http://www.math.pitt.edu/~bard/xpp/xpp.html), or Oscill8 (http://oscill8.sourceforge.net/). All data was then plotted using MATLAB (https://mathworks.com/).

## Electronic supplementary material

Supplementary information
