# Supplementary material for: A dynamical framework for complex fractional killing
Source: Sci Rep. 2017 Aug 14;7:8002. doi: 10.1038/s41598-017-07422-2 (PMC5556027; doi:10.1038/s41598-017-07422-2)

Supplementary information for the manuscript "***A dynamical framework for complex fractional killing***"

Richard Ballweg[1] Andrew L. Paek[2] and Tongli Zhang[1*]

[1] Department of Molecular and Cellular Physiology, College of Medicine, University of Cincinnati, Cincinnati, OH

[2] Department of Molecular and Cellular Biology, University of Arizona, Tucson, AZ

* Correspondence should be addressed to Tongli.zhang@uc.edu

Supplementary Figure 1. Effect of the level of noise on time series simulations. The integrated levels of p53 are shown. Red curves indicate apoptotic cells while blue curves indicate surviving cells. **A** & **B**, 300 cells were subjected to a low level of drug (drug=0.45), and the values of the chosen parameters were randomized between 60-140% (**A**) or 80-100% (**B**) of their basal values. **C** & **D**, 300 cells are subjected to a high level of drug (drug=1), and the parameter values are randomized 60-140% (**C**) or 80-100% (**D**).

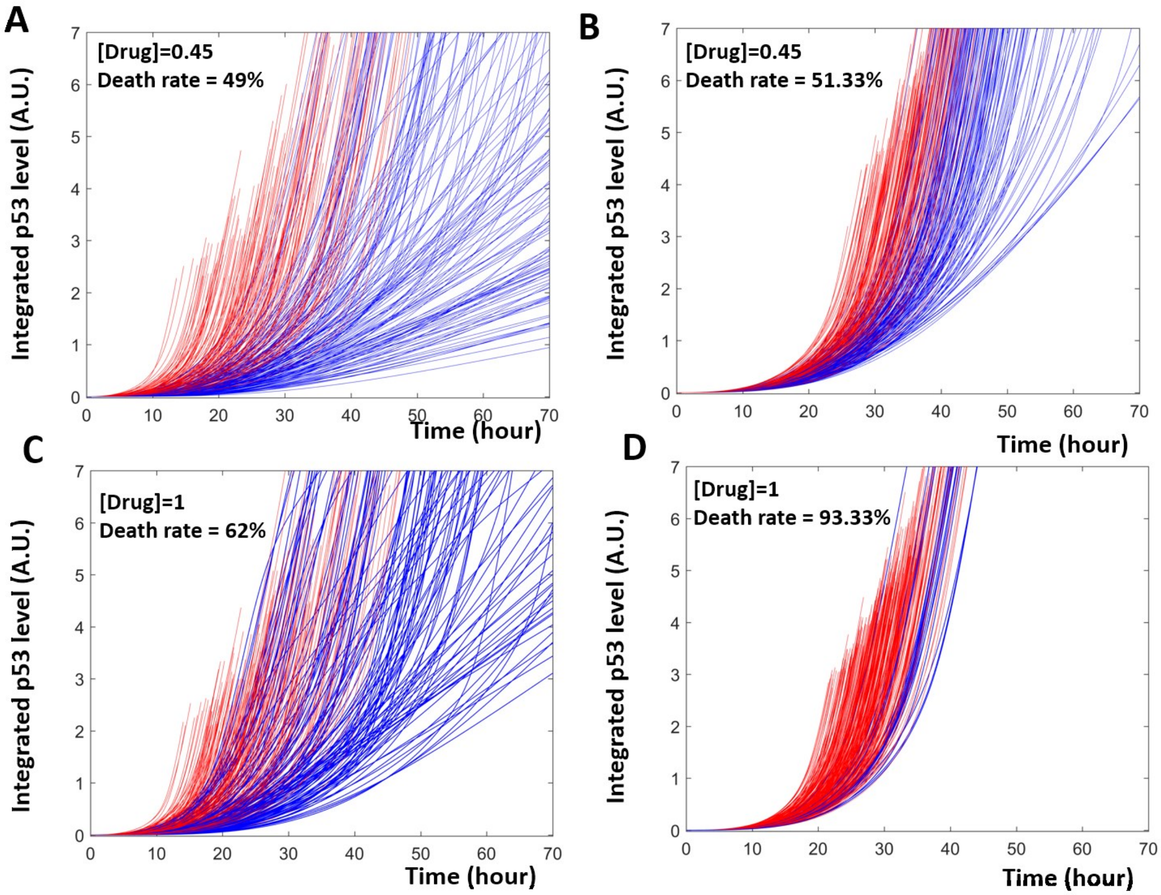

Supplement: Supplementary file 1 — Supplementary information [file 41598_2017_7422_MOESM1_ESM.pdf]
